# Pediatric Immunization Practices in Nephrotic Syndrome: An Assessment of Provider and Parental Knowledge

**DOI:** 10.3389/fped.2020.619548

**Published:** 2021-02-05

**Authors:** Cheryl L. Tran, David T. Selewski, Gia J. Oh, Jonathan P. Troost, Susan F. Massengill, Samhar I. Al-Akash, Shefali Mahesh, Rasheda Amin, Isa F. Ashoor, Rahul Chanchlani, Mahmoud Kallash, Robert P. Woroniecki, Debbie S. Gipson

**Affiliations:** ^1^Division of Pediatric Nephrology, Department of Pediatrics, Mayo Clinic, Rochester, MN, United States; ^2^Division of Pediatric Nephrology, Department of Pediatrics, Medical University of South Carolina, Charleston, SC, United States; ^3^Division of Nephrology, Department of Pediatrics, Stanford University, Stanford, CA, United States; ^4^Michigan Institute for Clinical and Health Research, University of Michigan, Ann Arbor, MI, United States; ^5^Levine Children's Hospital at Atrium Health, Charlotte, NC, United States; ^6^Driscoll Children's Hospital, Corpus Christi, TX, United States; ^7^Division of Nephrology and Dialysis, Akron Children's Hospital, Akron, OH, United States; ^8^Pediatric Specialists of Virginia, Fairfax, VA, United States; ^9^Department of Pediatrics, George Washington University, Washington, DC, United States; ^10^Division of Nephrology, Department of Pediatrics, Louisiana State University, New Orleans, LA, United States; ^11^Division of Pediatric Nephrology, Department of Pediatrics, McMaster University, Hamilton, ON, Canada; ^12^Division of Nephrology, Nationwide Children's Hospital, Columbus, OH, United States; ^13^Division of Pediatric Nephrology and Hypertension, Stony Brook Children's Hospital and Renaissance School of Medicine, Stony Brook, NY, United States; ^14^Division of Pediatric Nephrology, Department of Pediatrics, University of Michigan, Ann Arbor, MI, United States

**Keywords:** nephrotic syndrome, children, immunization, immunosuppression, education

## Abstract

**Background:** Children with nephrotic syndrome (NS) are at high risk for vaccine-preventable infections due to the immunological effects from the disease and concurrent treatment with immunosuppressive medications. Immunizations in these patients may be deferred due to their immunosuppressive treatment which may increase the risk for vaccine-preventable infections. Immunization practices in children with NS continue to vary among pediatric nephrologists. This raises the question of whether children with NS are receiving the recommended vaccinations at appropriate times. Therefore, it is critical to understand the practices and patient education provided by physicians to patients on the topic of vaccinations.

**Methods:** After informed consent, parents/guardians of 153 pediatric patients (<18 years old) diagnosed with NS from 2005 to 2018 and 50 pediatric nephrologists from 11 participating centers completed anonymous surveys to evaluate immunization practices among pediatric nephrologists, assess the vaccine education provided to families of children with NS, assess the parental knowledge of immunization recommendations, and assess predictors of polysaccharide pneumococcal vaccine adherence. The Advisory Committee on Immunization Practices (ACIP) Immunization 2019 Guideline for those with altered immunocompetence was used to determine accuracy of vaccine knowledge and practices.

**Results:** Forty-four percent of providers self-reported adherence to the ACIP guidelines for inactive vaccines and 22% to the guidelines for live vaccines. Thirty-two percent of parents/guardians reported knowledge that aligned with the ACIP guidelines for inactive vaccines and 1% for live vaccines. Subjects residing in the Midwest and provider recommendations for vaccines were positive predictors of vaccine adherence (*p* < 0.001 and *p* 0.02, respectively).

**Conclusions:** Vaccine recommendation by medical providers is paramount in vaccine adherence among pediatric patients with NS. This study identifies potential educational opportunities for medical subspecialty providers and family caregivers about immunization recommendations for immunosuppressed patients.

## Introduction

Nephrotic syndrome (NS) is caused by renal diseases that affect the permeability of the glomerular filtration barrier resulting in massive proteinuria, including immunoglobulins and complement proteins (1–4). Given the immunological effects from the disease and concurrent treatment with immunosuppressive medications, children with NS are at high risk for severe bacterial infections, especially with encapsulated bacteria (5). *Streptococcus pneumoniae* is a common encapsulated bacterial pathogen that is known to cause serious infections in children with NS (6, 7). Given the risk for this infectious pathogen, the pneumococcal polysaccharide vaccine (PPSV 23) has been specifically recommended for pediatric patients with certain medical conditions/diseases (i.e., NS, chronic renal failure, and immunosuppression medications). This susceptibility to infection extends beyond bacterial infections and includes serious viral infections such as Varicella, which can result in severe disseminated infections in immunocompromised hosts.

Children with NS have a high burden of healthcare utilization with a mean charge per hospitalization of $26,500 that exceeds many other chronic illnesses (8). Gipson et al. evaluated hospitalization costs in a cohort of children with NS and clearly showed that serious complications of NS, including infection, occur commonly and increase healthcare costs. In this study, 16% of 9,934 discharges in 2006 and 2009 had at least one severe complication (pneumonia, sepsis, peritonitis, thromboembolism, or diabetes) attributable to NS or its treatment. Infection-related complications were the most common including pneumonia, sepsis, or peritonitis. In 2019, Carpenter et al. investigated the prevalence of infection and venous thromboembolism in hospitalized pediatric patients with NS (9). This group demonstrated high rates of infection in hospitalized pediatric NS patients, with *Streptococcus pneumoniae* being the most common pathogen. Appreciating the infectious susceptibility associated with nephrotic syndrome is critical since improved vaccination practices would potentially aid in the prevention of many of these infections (10, 11).

This raises the question of whether children with NS are receiving the recommended vaccinations at appropriate times. A critical first step in this process is to understand the practices and patient education provided by physicians to patients on the topic of vaccinations. The aims of this are: (1) evaluate the immunization practices among pediatric nephrologists, (2) assess the education provided to families of children with NS by the pediatric nephrology providers, (3) assess the parental knowledge and understanding of these immunization recommendations, and (4) assess predictors of PPSV 23 adherence.

## Methods

Parents/guardians of pediatric patients (<18 years old) diagnosed with NS from 2005 to 2018 from 11 institutions in the Pediatric Nephrology Research Consortium and Kidney Research Network participated in the study. Patient inclusion criteria were children <18 years old with primary NS diagnosed between 2005 and 2018 who were seen in the pediatric nephrology practice at the participating center on at least one occasion. All pediatric nephrologists at the participating study centers were also included in the study. A parent/guardian survey was distributed, and a separate pediatric nephrology provider survey was distributed to pediatric nephrologists at each center. This study was performed in line with the principles of the Declaration of Helsinki. Institutional Review Boards of each participating center approved the study. Consent and assent were obtained per institutional guidelines.

### Survey and Measures

The parent/guardian survey contained 24 questions which were created by the authors and included demographic data, immunosuppression history, parental knowledge of immunization recommendations and their intent to follow the recommendations, and potentially vaccine-preventable hospitalizations for infections. The provider survey contained 21 questions which were created by the authors and included demographic data, provider immunization recommendation practices, knowledge of the current immunization guidelines, and hospitalizations for infections at any time during their practice that may have been vaccine-preventable. Family caregivers/study coordinators provided redacted copies of patient immunization records. Validation studies of self-administered surveys have shown this type of survey methodology to be a promising tool across medical disciplines (12–14). Surveys were self-administered by the families and providers in the clinic office. The surveys are provided in the Supplementary Material 1, 2.

**Figure 1:**
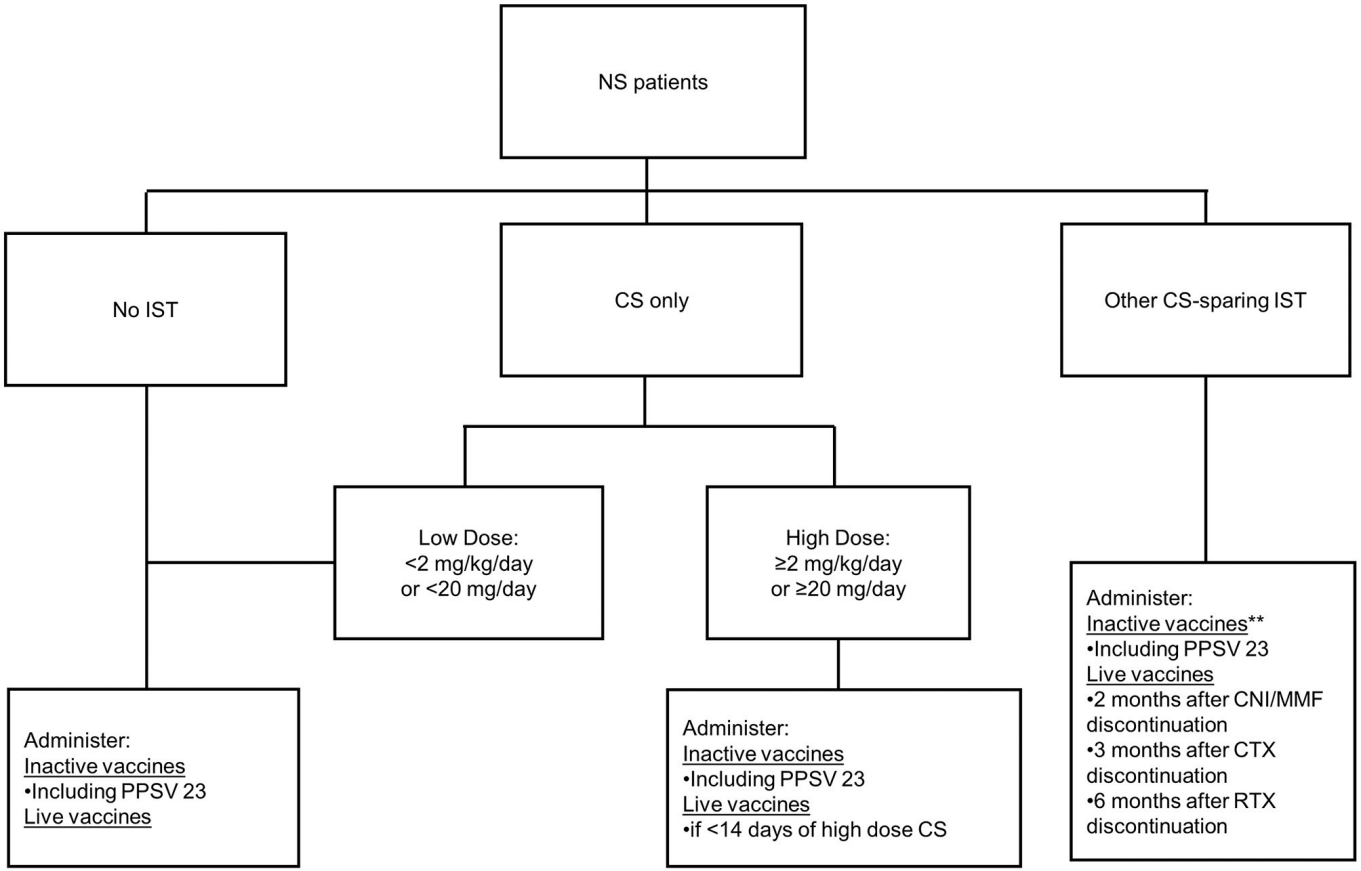
Flow diagram of abbreviated vaccine guidelines. Information for this was adapted from the Advisory Committee on Immunization Practices 2019 vaccine guidelines. Abbreviations: NS, nephrotic syndrome; IST, immunosuppression; CS, corticosteroids; PPSV 23, polysaccharide pneumococcal vaccine; CNI, calcineurin inhibitor; MMF, mycophenolate mofetil; CTX, cyclophosphamide; RTX, rituximab. **Defer 6 months after RTX discontinuation, or administer if no finite endpoint for RTX and assess titers after discontinuation of RTX.

The Advisory Committee on Immunization Practices (ACIP) Recommended Child and Adolescent Immunization Schedule was used to determine accuracy of parent and provider knowledge of current immunization guidelines (Figure 1) (15). In brief summary, the ACIP recommends inactive vaccines for any pediatric patient who is not receiving immunosuppression medications as well as those on most immunosuppression therapies. There is an exception to consider deferring inactive vaccines for 6 months after receiving B-cell depleting therapy, or may consider administration of inactive vaccines while receiving B-cell depleting therapy with assessment of titers after discontinuation of the B-cell depleting agent. With regards to live vaccines, the ACIP recommends administration of live vaccines in patients who are not on immunosuppression therapy; patients who have received <14 days of high dose corticosteroids; and patients who have been off of Calcineurin inhibitors (CNI)/Mycophenolate Mofetil (MMF) for 2 months, off of cytotoxic agents (i.e., Cyclophosphamide) for 3 months, and off of B-cell depleting agents (i.e., Rituximab) for 6 months. A more detailed summary of this guideline is provided in Supplementary Material 3.

Patient demographic variables included state of residence, patient age, race, parent/guardian level of education, and household income. Provider demographic variables included institution, type of practice (academic vs. private), type of subspecialty training (Pediatric Nephrology, Internal Medicine-Pediatric Nephrology, or Internal Medicine Nephrology), and percent effort allocated to clinical care.

### Statistical Analysis

Descriptive statistics included frequencies for categorical variables and mean ± standard deviation (SD) or median (IQR) for continuous variables, as appropriate. Fischer's exact tests were used to compare knowledge and guidelines across regions, and evaluate predictors of PPSV 23 vaccination. Multivariable logistic regression was used to test independent predictors of PPSV 23 vaccination. Variables with unadjusted *p* < 0.2 entered a multivariable model and were removed in descending order until all remaining terms were significant. For all analyses, *p* ≤ 0.05 were considered statistically significant.

## Results

### Study Population Demographics

Between November 2015 and December 2018, 175 pediatric patients diagnosed with NS between January 2005 and December 2018, from 11 North American centers, were eligible for the study, of which 153 parents/guardians of pediatric subjects consented to the study and completed the anonymous survey (parent/guardian response rate 87.4%). Fifty-eight pediatric nephrologists at participating centers were provided an anonymous survey, of which 50 (86.2%) completed the survey. All subjects were stratified by region [Coastal (3 centers), Midwest (4 centers), and South (4 centers)]. Due to the small number of study centers on either coast, the current study grouped these centers into one “Coastal” region to avoid identifying a single site and its study participants.

At study enrollment (Table 1a), the median age at NS diagnosis was 3 years of age (IQR 2-5); nearly half of the pediatric patients (46%) were 6–11 years of age at study enrollment; 40% were White; majority of parents/guardians had some post high school education (77%); and nearly all pediatric patients had exposure to immunosuppression (99%). In the pediatric nephrology provider population (Table 1b), the majority practiced in an academic setting (86%); majority trained specifically in Pediatric Nephrology (94%); and all provided care to pediatric patients with the median percent effort allocated to clinical care of 70% (IQR 55–86%).

**Table 1a:** Demographic description of pediatric nephrotic syndrome subject population.

**Variable**	**NS subject study participants *n* = 153**
**Current Age (y), n (%)**	
0–5	46 (30)
6–11	70 (46)
12–22	37 (24)
**Age at Diagnosis (y), n (%)**	
0–5	117 (76)
6–11	30 (20)
12–18	6 (4)
**Race, n (%)**	
Asian/Pacific Islander	21 (14)
Black/African American	27 (18)
Hispanic/Latino	25 (16)
Native American	4 (3)
White/Caucasian	62 (40)
Other	14 (9)
**Region of care, n (%)**	
Coastal	28 (18)
Midwest	64 (42)
South	61 (40)
**Highest level of parent education, n (%)**	
No schooling to 8th grade	3 (2)
Some high school to high school graduate	30 (20)
Some college, college graduate, or vocational training	87 (57)
Some postgraduate work to postgraduate degree	30 (20)
Decline to answer	3 (2)
**Household annual income, n (%)**	
< $25,000	32 (21)
$25,000–$39,999	22 (14)
$40,000–$49,999	7 (5)
$50,000–$74,999	15 (10)
$75,000–$99,999	20 (13)
>$100,000	44 (29)
Decline to answer	13 (8)
**Immunosuppression exposure, n (%)**	
Calcineurin inhibitor	94 (61)
Cyclophosphamide	19 (12)
Mycophenolate mofetil	55 (36)
Prednisone	143 (93)
Rituximab	23 (15)
Other	3 (2)
None	2 (1)
**Number of times seen by pediatric nephrology provider in the past year**	
Once	9 (6)
2–3 times	63 (41)
≥4 times	81 (53)

**Table 1b:** Demographic description of pediatric nephrology provider population.

**Variable**	**Pediatric nephrology provider participants *n* = 50**
**Region, n (%)**	
Coastal	12 (24)
Midwest	21 (42)
South	17 (34)
**Type of practice, n (%)**	
Academic	43 (86)
Private	4 (8)
Both academic & private	3 (6)
**Type of subspecialty training, n (%)**	
Pediatric nephrology	47 (94)
Internal medicine-pediatric nephrology	3 (6)
**Provider percent effort, median (IQR)**	
Clinical care to pediatric patients	70 (55–86)
Clinical care to adult patients	0 (0)
Administration	4 (0–20)
Research	5 (0–16)
Education	5 (0–10)

### Pediatric Nephrology Provider Vaccine Recommendations

Table 2 displays the pediatric nephrology provider recommendations for inactive vaccines. All providers indicated that they provide recommendations for inactive vaccines to patients. Eighty-two percent recommended inactive vaccines while off of steroids and in remission, and 78% recommended inactive vaccines on low dose steroids. However, only 44% recommended inactive vaccines while on high dose steroids with 58% recommending inactive vaccines when off of other immunosuppressive medications. Only 44% recommended inactive vaccines at any time regardless of immunosuppression status. Overall, 56% of providers had at least one response inconsistent with guidelines.

**Table 2:** Provider inactive vaccine recommendation.

**Inactive immunizations**	**Total** ***N* = 50 (%)**	**Coastal** ***N* = 12 (%)**	**Midwest** ***N* = 21 (%)**	**South** ***N* = 17 (%)**	**Fischer's** ** exact *p***
I do not provide recommendations regarding INACTIVE immunizations	0 (0)	0 (0)	0 (0)	0 (0)	—
**I provide/recommend when the patient is…**					
a. Off steroids and in remission	41 (82)	10 (83)	16 (76)	15 (88)	0.73
b. On low dose (every other day) steroids	39 (78)	10 (83)	17 (81)	12 (71)	0.75
c. On daily high dose steroids	22 (44)	5 (42)	7 (33)	10 (59)	0.28
d. Off other immunosuppressive medications (i.e., cyclosporine, tacrolimus, mycophenolate mofetil, cyclophosphamide, rituximab)	29 (58)	9 (75)	9 (43)	11 (65)	0.18
I would not alter the immunization schedule for INACTIVE immunizations in patients with NS. They can receive them at any time	22 (44)	5 (42)	7 (33)	10 (59)	0.28
I would never provide an INACTIVE immunization to a NS patient regardless of therapy or remission status	0 (0)	0 (0)	0 (0)	0 (0)	—
All responses consistent with guidelines	22 (44)	5 (42)	7 (33)	10 (59)	0.28

*Data are shown as number (percent)*.

*NS, nephrotic syndrome*.

Table 3 displays the pediatric nephrology provider recommendations for live vaccines. Of the 50 providers surveyed, only 1 (2%) indicated they did not provide live vaccine recommendations to patients. Seventy-eight percent recommended live vaccines while off steroids and in remission; however, only 30% recommended live vaccines while on low dose steroids. All providers recommended avoiding live vaccines while on daily high dose steroids, and 76% recommended receiving live vaccines while off other immunosuppressive medication. Overall, only 22% of provider's responses were in complete agreement with the ACIP guidelines for live vaccines. The main deviance from the ACIP guidelines was with respect to low dose steroids. Of the 39 providers that did not follow guidelines, 35 (90%) indicated they would not recommend live vaccines while on low dose steroids.

**Table 3:** Provider live vaccine recommendation.

**Live immunizations**	**Total** ***N* = 50 (%)**	**Coastal** ***N* = 12 (%)**	**Midwest** ***N* = 21 (%)**	**South** ***N* = 17 (%)**	**Fischer's** ** exact *p***
I do not provide recommendations regarding LIVE immunizations	1 (2)	0 (0)	1 (5)	0 (0)	0.99
**I provide/recommend when the patient is…**					
a. Off steroids and in remission	39 (78)	9 (75)	17 (81)	13 (76)	0.91
b. On low dose (every other day) steroids	15 (30)	3 (25)	7 (33)	5 (29)	0.93
c. On daily high dose steroids	0 (0)	0 (0)	0 (0)	0 (0)	—
d. Off other immunosuppressive medications (i.e., cyclosporine, tacrolimus, mycophenolate mofetil, cyclophosphamide, rituximab)	38 (76)	10 (83)	15 (71)	13 (76)	0.91
I would not alter the immunization schedule for LIVE immunizations in patients with NS. They can receive them at any time	0 (0)	0 (0)	0 (0)	0 (0)	—
I would never provide a LIVE immunization to a NS patient regardless of therapy or remission status	0 (0)	0 (0)	0 (0)	0 (0)	—
All responses consistent with guidelines	11 (22)	3 (25)	5 (24)	3 (18)	0.83

*Data are shown as number (percent)*.

*NS, nephrotic syndrome*.

### Pediatric NS Parent/Guardian Vaccine Knowledge

Table 4 shows the parent/guardian knowledge of recommended inactive vaccines. Out of 153 respondents, 13% indicated that no one told them when it was acceptable to receive inactive immunizations. Of the remaining, only 48% indicated that inactive vaccines could be administered while off steroids and in remission with 38% indicating that inactive vaccines could be given on low dose steroids. Only 33% indicated that inactive vaccines could be administered while on high dose steroids with 33% indicating that inactive vaccines could be given when off other immunosuppressive medications. Overall, 32% of parents/guardian responses suggested understanding of immunization practices that aligned with ACIP guidelines for inactive vaccines.

**Table 4:** Parent inactive vaccine recommendation.

**Inactive immunizations**	**Total** ***N* = 153 N (%)**	**Coastal** ***N* = 28 N (%)**	**Midwest** ***N* = 64 N (%)**	**South** ***N* = 61 N (%)**	**Fischer's** ** exact *p***
No one ever told me when it was ok to receive INACTIVE immunizations.	20 (13)	6 (21)	4 (6)	10 (16)	0.07
**I was told it was OK to receive INACTIVE immunizations when my child is…**					
a. Off steroids and in remission	74 (48)	12 (43)	39 (61)	23 (38)	0.03
b. On Low dose (every other day) steroids	58 (38)	8 (29)	30 (47)	20 (33)	0.15
c. On daily high dose steroids	50 (33)	8 (29)	24 (38)	18 (30)	0.57
d. Off other immunosuppressive medications (i.e., cyclosporine, tacrolimus, mycophenolate mofetil, cyclophosphamide, rituximab)	51 (33)	7 (25)	24 (38)	20 (33)	0.56
I was told that my child could receive INACTIVE immunizations at any time regardless of type of medication or remission status.	49 (32)	7 (25)	24 (38)	18 (30)	0.47
I was told that my child could never receive INACTIVE immunizations.	0 (0)	0 (0)	0 (0)	0 (0)	—
Parent had correct knowledge of guidelines	49 (32)	7 (25)	24 (38)	18 (30)	0.47

*Data are shown as number (percent)*.

Table 5 shows the parent/guardian knowledge of recommended live vaccines. Twenty percent of parents/guardians indicated that no one told them when it was acceptable to receive live immunizations. Of the remaining, 27% indicated that live vaccines could be administered while off steroids and in remission; 8% indicated that live vaccines could be given while on low dose steroids; and 4% of parents/guardians indicated that live vaccines could be administered on high dose steroids. Twenty-one percent indicated that live vaccines could be administered when off other immunosuppression medications. Overall, 1% of parent/guardian responses suggested an understanding of current live vaccination best practices.

**Table 5:** Parent live vaccine recommendation.

**Live immunizations**	**Total** ***N* = 153 N (%)**	**Coastal** ***N* = 28 N (%)**	**Midwest** ***N* = 64 N (%)**	**South** ***N* = 61 N (%)**	**Fischer's** ** exact *p***
No one ever told me when it was ok to receive LIVE immunizations.	30 (20)	10 (36)	9 (14)	11 (18)	0.05
**I was told it was OK to receive LIVE immunizations when my child is…**					
a. Off steroids and in remission	41 (27)	6 (21)	21 (33)	14 (23)	0.37
b. On low dose (every other day) steroids	12 (8)	0 (0)	9 (14)	3 (5)	0.05
c. On daily high dose steroids	6 (4)	0 (0)	4 (6)	2 (3)	0.56
d. Off other immunosuppressive medications (i.e., cyclosporine, tacrolimus, mycophenolate mofetil, cyclophosphamide, rituximab)	32 (21)	2 (7)	21 (33)	9 (15)	0.007
I was told that my child could receive LIVE immunizations at any time regardless of type of medication or remission status.	5 (3)	0 (0)	4 (6)	1 (2)	0.27
I was told that my child could never receive LIVE immunizations.	18 (12)	5 (18)	7 (11)	6 (10)	0.57
Parent had correct knowledge of guidelines	1 (1)	0 (0)	0 (0)	1 (2)	0.58

*Data are shown as number (percent)*.

### Pediatric NS Patient Immunization Status Validation and Predictors

One-hundred and 52 parents/guardians of pediatric NS subjects (99%) indicated that their child had been vaccinated and one pediatric NS subject (1%) had never been vaccinated. Records of vaccinations were available from 141 (93%) participants for validation of immunization status. Immunization status was considered up to date if the subject received all ACIP recommended vaccines based on immunosuppression status, age at time of survey, and vaccine release date. One-hundred and twenty-two subjects (87%) were up to date on the recommended vaccines. Fifty-three percent received the 7-valent pneumococcal vaccine, 71% received the 13-valent pneumococcal vaccine, and 48% received the PPSV 23.

Subjects residing in the Midwest was a positive predictor of subjects receiving the PPSV 23 (*p* < 0.001). Additionally, receiving immunization recommendations from the pediatric nephrology provider was a positive predictor of subjects receiving the PPSV 23 as well (*p* 0.02) (Table 6). Multivariable logistic regression of the differences in Table 6 revealed that region and immunization recommendations were independent predictors of vaccinations. Those in the Coastal region were less likely to be vaccinated than the Midwest (OR = 0.30 95% CI = 0.13–0.66) and those with recommendations from the pediatric nephrologist were more likely to be vaccinated than those without recommendations (OR = 1.95 95% CI = 1.05–3.63) (Table 7). When provider responses were reviewed, providers in the Coastal region were less likely to review immunization records on a periodic basis (*p* < 0.01) and were more likely to state that they were unsure if they would recommend the PPSV23 if the patient had previously received the 7-valent pneumococcal conjugate vaccine (PCV) or PCV-13 (*p* 0.03) (Supplementary Material 4).

**Table 6:** Pediatric NS patient PPSV 23 vaccine adherence and predictors^*^.

**Variable**	**N (% NS children vaccinated to PPSV 23)**	**N (% NS children unvaccinated to PPSV 23)**	**Fischer's exact *p***
Parent income (*N* = 125)			0.30
< $25,000 (*n* = 29)	17 (58.6)	12 (41.4)	
$25,000–39,999 (*n* = 19)	8 (42.1)	11 (57.9)	
$40,000–49,999 (*n* = 7)	14 (57.1)	3 (42.9)	
$50,000–74,999 (*n* = 15)	11 (73.3)	4 (26.7)	
$75,000–99,999 (*n* = 18)	10 (55.6)	8 (44.4)	
≥$100,000 (*n* = 37)	15 (40.5)	22 (59.4)	
Parent education (*N* = 134)			0.07
No schooling to 8th grade (*n* = 2)	2 (100)	0 (0)	
Some high school to high school graduate (*n* = 25)	10 (40)	15 (60)	
Some college, college graduate, or vocational training (*n* = 82)	47 (57.3)	35 (42.7)	
Some postgraduate work to postgraduate degree (*n* = 25)	9 (36)	16 (64)	
Race (*N* = 135)			0.83
White (*n* = 53)	28 (52.8)	25 (47.1)	
Black (*n* = 25)	12 (48)	13 (52)	
Hispanic (*n* = 24)	14 (58.3)	10 (41.7)	
Asian (*n* = 17)	8 (47.1)	9 (52.9)	
Native American (*n* = 4)	1 (25)	3 (75)	
Other (*n* = 12)	5 (41.7)	7 (58.3)	
Region (*N* = 135)			<0.001
Coastal (*n* = 17)	1 (5.9)	16 (94.1)	
Midwest (*n* = 58)	38 (65.5)	20 (34.4)	
South (*n* = 60)	29 (48.3)	31 (51.7)	
Number of times seen by pediatric nephrology provider in the past year (*N* = 135)			0.99
Once	4 (44.4)	5 (55.6)	
2–3 times	28 (50.9)	27 (49.1)	
≥4 times	36 (50.7)	35 (49.3)	
Immunization recommendations provided by Pediatric nephrologist (*N* = 118)			0.02
Yes (*n* = 100)	55 (55)	45 (45)	
No (*n* = 18)	4 (22.2)	14 (77.8)	

*NS, nephrotic syndrome; PPSV 23, polysaccharide pneumococcal vaccine 23-valent*.

* *Excluded the following from analysis: (1) Centers in Coastal region without vaccine records to validate PPSV 23 status, (2) Pediatric NS subjects who were unable to receive the PPSV 23 due to age eligibility, (3) Parents/guardians who declined to answer in the parent income and parent education variables, and (4) Parents/guardians who answered “I am not sure” in Immunization recommendations provided by pediatric nephrologist variable*.

**Table 7:** Adjusted logistic regression model of PPSV 23 vaccine (Complete case analysis *n* = 110).

**Variable**	**Odds ratio [95% confidence interval]**	***p*-value**
Region		0.01
Coastal	0.30 [0.13–0.66]	0.003
Midwest	Reference	Reference
South	0.56 [0.24–1.30]	0.18
Immunization recommendations provided by pediatric nephrologist		0.04
Yes	1.95 [1.05–3.63]	0.04
No	Reference	Reference

*PPSV 23, polysaccharide pneumococcal vaccine 23-valent*.

## Discussion

While there have been published studies investigating vaccine practices and vaccine adherence patterns, these studies have evaluated the medical provider population alone or the parent population alone. Our study investigates both the subspecialty medical provider and parent population from the same institution and region simultaneously. This study demonstrates gaps in vaccine knowledge of both pediatric nephrology providers and parents of children with NS who are immunosuppressed.

The decision to vaccinate a child can be influenced by parental knowledge, parental understanding of the benefits of vaccination, provider knowledge, and other complex factors related to their child's underlying disease and treatment. Deficiencies in vaccinations directly impact the health of the individual child and of the larger pediatric community if herd protection is not achieved due to poor vaccine adherence. Children most at risk for severe vaccine-preventable infections are those who are immunocompromised, which includes children with NS.

The Center for Disease Control (CDC), ACIP, and the Committee on Infectious Diseases of the American Academy of Pediatrics (AAP) release an updated recommended immunization schedule for healthy children annually (16). This immunization schedule is available to medical providers online (http://www.cdc.gov/vaccines/recs/schedules/child-schedule.htm). In addition to the yearly updated recommendations, health care providers should be aware of guidelines for vaccinating immunocompromised patients, which are readily available online (https://www.cdc.gov/vaccines/hcp/acip-recs/general-recs/immunocompetence.html and https://www.cdc.gov/vaccines/schedules/downloads/child/0-18yrs-child-combined-schedule.pdf). Despite these guidelines and published comprehensive reviews outlining vaccine guidelines in various immunosuppressed states, there remains a discrepancy in vaccine practices among pediatric nephrologists in the management of children with NS on immunosuppressive therapy (17–19).

The discrepancy in vaccine practice amongst providers and low compliance in following ACIP guidelines is likely multifactorial. It could be in part due to the provider's misinterpretation of vaccine guidelines due to the complex immunosuppression regimens that these NS patients may require, or the provider's disagreement of vaccination guidelines based on anecdotal experience with NS relapses and/or belief that immunosuppressed patients may not mount a robust vaccine response. In this study, only 44% of provider responses were in complete alignment with the ACIP guidelines for inactive vaccines and 22% for live vaccines. The CDC general principles for those with altered immunocompetence, state that inactivated vaccines could be deferred during a time of immunosuppression since vaccines may be less effective during this period. However, if an inactivated vaccine is given during the time that a patient is immunosuppressed, then the inactive vaccine may need to be repeated when immune function has improved. Yet, the CDC guidelines emphasize the need for immunocompromised patients to receive certain inactive conjugated and polysaccharide-based vaccines (i.e., pneumococcal vaccines, *Haemophilus influenzae* type b, and meningococcal vaccines) due to the increased risk for disease if the vaccine is withheld. Live vaccines are typically deferred until immune function has improved due to the risk of uninhibited growth of the attenuated live virus or bacteria in those with altered immunocompetence (20–23). It is clear that the vaccine guidelines for those who are immunosuppressed can be nuanced.

Since 80% of pediatric patients with new onset NS are steroid-sensitive and eventually taper off of steroid therapy, some pediatric nephrologists may opt to withhold vaccines until these patients have return of immune function to help mount a more effective response to the vaccine (24). In our study, 44% of the pediatric nephrologists surveyed reported withholding inactive vaccines when a NS patient is on any dose of steroids due to concern that the patient may not mount an immune response. Ten percent of the pediatric nephrologists surveyed observed a NS relapse after immunization administration. However, published studies have demonstrated vaccine efficacy in NS patients who are immunosuppressed. Aoun and Ulinski exhibited good serologic response to the PPSV 23 in children with NS on high dose prednisone in the short term and in the long term (25). They also demonstrated that the long term pneumococcal antibody response was not impacted by other immunosuppressive agents (26). Hsu et al. demonstrated good vaccine efficacy of the pneumococcal vaccine in children with NS on high dose steroids and no differences in rate of NS relapse (27). In addition, other inactive polysaccharide-based vaccines, such as meningococcal C conjugate vaccine has not been shown to be associated with increased NS relapses (28). Other studies have demonstrated that the Varicella vaccine has been well-tolerated in children with NS and highly immunogenic in those on low-dose, every other day prednisone (29, 30). Despite these groups demonstrating vaccine efficacy without differences in NS relapse rates, there has been a wide variation of vaccine practices as evidenced by two studies conducted in 1993 and 2001 (17, 18). Our study similarly observed varied vaccine practices by providers, which translates to the gap in parental knowledge and understanding of the timing of vaccine administration in their children with NS as seen in the parental/guardian survey responses.

NS can be a difficult disease to treat especially in patients who have a steroid-dependent, frequently-relapsing, or steroid-resistant clinical course. These patients may have no or very brief periods of time off of immunosuppression medications to keep the disease in control. As a result, opportunities to vaccinate with inactive and/or live vaccines become limited if providers choose to withhold immunizations until full recovery of immune function. Withholding vaccines in this high-risk population can lead to an increased risk of vaccine-preventable disease (9). It is known that infection is a common trigger for NS relapses which can result in the need for hospitalization to help manage the complications of the relapse, thus leading to significantly higher hospitalization charges (8).

Factors associated with immunization adherence have been studied by various groups. Our group specifically evaluated the adherence to PPSV23 administration since it is recommended by the ACIP for pediatric patients with NS and immunosuppressed states. Cost of vaccines, lack of parental knowledge of the benefits of vaccines, and lack of vaccine recommendation by medical providers has been reported to adversely impact immunization adherence (31, 32). Our study similarly showed that provider vaccine recommendations were associated with vaccine adherence to PPSV 23. However, we did not investigate the cost of vaccines as a potential factor of immunization adherence in this survey study. Schuller et al. demonstrated an increased likelihood of vaccination if the child's caregiver received higher education, lived in the Northeast, had private insurance, and was of Hispanic race among US children (33). We found that subjects residing in the Midwest were more likely to receive the PPSV 23, which may be due to a higher number of participating study sites in the Midwest region compared to other regions surveyed in North America for our study, and therefore the Midwest region may be more representative than a region with fewer sites. Additionally, parent income, level of parent education, and race were not positive predictors of subjects receiving the PPSV 23 in the current study. The differences in the factors associated with vaccines adherence in our study compared to other published studies may also be explained by our small sample size. Our study also evaluated other possible contributing factors to vaccine adherence including the ability of the subspecialty clinic to provide vaccines (i.e., pneumococcal vaccine) and the communication of vaccine recommendations from the pediatric nephrology provider to the patient's primary care provider (PCP). Seventy-six percent of providers indicated that their subspecialty clinic can administer pneumococcal vaccines. The remaining providers who do not have this capability may defer the responsibility of vaccinations to the PCP. Ninety-two percent of providers indicated that they communicate their vaccine recommendations to their patient's PCP with a letter as the most common modality of communication.

The current study demonstrates the need for a more uniform vaccine practice in the pediatric NS population among pediatric nephrologists, which will in turn better educate and help parents/guardians understand the importance of vaccination and when it is appropriate for their child to receive certain vaccines.

### Limitations and Strengths

The limitations of this study include the heterogeneity of survey responses among the parent/guardian cohort and pediatric nephrology providers with regards to knowledge of the ACIP guidelines. The discrepant responses amongst both groups could be explained by comprehension and communication between parent and provider or alternatively by survey interpretation. Since the surveys were anonymous for both cohorts, we were unable to associate the parent/guardian and provider response resulting in the inability to directly correlate responses. While this study evaluated the communication of vaccine recommendations from pediatric nephrologist to PCP, an assessment of the knowledge and practice of the patient's PCP was not evaluated which could have identified another area for improvement of vaccine adherence in this cohort. Lastly, the small sample size of this study may have played a role in the differences in factors associated with vaccine adherence compared to other published studies.

The strengths of this study include the high response rate. This study also investigated the immunization knowledge from both pediatric nephrology providers and parent/guardians of children with NS simultaneously from their shared institutions, which helped to evaluate for any institution/region-specific immunization practices.

## Conclusion

Our findings support the growing evidence that vaccine recommendation by medical providers is paramount in vaccine adherence among pediatric patients with NS. The disparate survey responses among the pediatric nephrology providers and parents/guardians likely reflect the individual interpretations of the ACIP guidelines by the medical providers and possibly their anecdotal experiences with treatment of NS. Additionally, some pediatric nephrology practices do not have the capability to administer certain vaccines (i.e., PPSV 23) and therefore rely on vaccinations through the patient's PCP. Ensuring primary care establishment with a PCP as well as being a champion to clarify and communicate vaccination provisioning guidance with the patient's PCP may help to improve vaccine adherence. Future studies assessing the knowledge and practice of vaccine recommendations by PCPs for pediatric patients with NS may also highlight other areas for improvement of vaccine adherence such as communication between healthcare providers to assure vaccinations occur in a timely and complete fashion in the child's primary care office. Lastly, it is important that the ACIP immunization guidelines are reviewed by the pediatric nephrologist and primary care physicians yearly to be informed of any change in the immunization practice recommendations for those with altered immune competence.

## Data Availability Statement

The original contributions presented in the study are included in the article/Supplementary Material, further inquiries can be directed to the corresponding author/s.

## Ethics Statement

The studies involving human participants were reviewed and approved by each participating center. Written informed consent to participate in this study was provided by the participants' legal guardian/next of kin.

## Author Contributions

Data analysis was performed by JT and CT. All authors read and approved the final manuscript, contributed to the study conception, design, and data collection.

## Conflict of Interest

The authors declare that the research was conducted in the absence of any commercial or financial relationships that could be construed as a potential conflict of interest.

## Abbreviations

ACIP: Advisory Committee on Immunization Practices
AAP: American Academy of Pediatrics
CDC: Center for Disease Control
NS: nephrotic syndrome
PPSV 23: pneumococcal polysaccharide vaccine
PCP: primary care provider.
