# Supplementary material for: Pediatric Immunization Practices in Nephrotic Syndrome: An Assessment of Provider and Parental Knowledge
Source: Front Pediatr. 2021 Feb 5;8:619548. doi: 10.3389/fped.2020.619548 (PMC7901920; doi:10.3389/fped.2020.619548)
Supplement: Supplementary file 1 [file Table_1.DOCX]

**Supplement 1**

Nephrotic Syndrome (NS) Immunization survey (PROVIDER SURVEY)

Today’s date: ______________________________

DEMOGRAPHICS:

1. What is the name of your institution?
   1. List name of institution __________________________________________________
   2. I decline to respond.
2. Where is your practice located (List the state)?______________________________________
3. What is your type of practice?
   1. Academic
   2. Private
4. What type of subspecialty training did you have?
   1. Pediatric Nephrology
   2. Med-Peds Nephrology
   3. Internal Medicine Nephrology
5. What percent effort are you providing in the following areas? (Should add up to 100%)
   1. Clinical care to pediatric patients ________(%)
   2. Clinical care to adult patients ___________(%)
   3. Administration ______________________(%)
   4. Research ___________________________(%)
   5. Education ___________________________(%)

IMMUNIZATIONS:

1. How often do you/your staff review immunization records at **initial** evaluation of patients with nephrotic syndrome (NS)?
   1. Never
   2. Rarely
   3. Sometimes
   4. Often
   5. Always
   6. I am not sure
2. How often do you/your staff review immunization records on a **periodic basis (annually)** with your NS patients?
   1. Never
   2. Rarely
   3. Sometimes
   4. Often
   5. Always
   6. I am not sure
3. Do you routinely discuss with your nephrotic syndrome (NS) patients when they can receive certain immunizations and which immunizations they should receive?
   1. Yes
   2. No
   3. I am not sure
4. Do you feel that administering immunizations to NS patients in remission will result in a NS flare?
   1. Yes
      1. If yes, then have you seen this occur in your NS patients? (Please circle) Yes or No.
   2. No
   3. I am not sure

Please answer Question 10 pertaining to **LIVE** immunizations (ie MMR, VZV, Flumist). **Circle all that apply.**

1. I provide/recommend **LIVE** immunizations to pediatric patients with NS when the patient is:
   1. I do not provide recommendations regarding LIVE immunizations
   2. Off steroids and in remission
   3. On low dose (every other day) steroids
   4. On daily high dose steroids
   5. Off other immunosuppressive medications (ie cyclosporine, tacrolimus, mycophenolate mofetil, cyclophosphamide, rituximab).
      1. How long off other immunosuppressive medications?
         1. Immediately off other immunosuppressive medications
         2. 1 month
         3. 2 months
         4. 3 months
         5. Other: _________________
   6. I would not alter the immunization schedule for LIVE immunizations in patients with NS. They can receive them at any time.
   7. I would never provide a LIVE immunization to a NS patient regardless of therapy or remission status.
   8. Other: _________________
2. Do you routinely check VZV IgG or titers in pediatric patients with NS?
   1. Yes
   2. No
   3. I am not sure

12) If VZV titers/IgG are low and the patient only received 1 dose of VZV, would you provide the 2^nd^ VZV dose?

1. Yes
2. No
3. Other: ­­­­­­­­­­­­­­­­­­________________

Please answer Question 12 pertaining to **INACTIVATED** immunizations (ie pneumococcal & inactivated influenza). **Circle all that apply.**

1. I provide/recommend INACTIVATED immunizations to pediatric patients with NS when the patient is:
   1. I do not provide recommendations regarding INACTIVATED immunizations
   2. Off steroids and in remission
   3. On low dose (every other day) steroids
   4. On daily high dose steroids
   5. Off other immunosuppressive medications (ie cyclosporine, tacrolimus, mycophenolate mofetil, cyclophosphamide, rituximab).
      1. How long off other immunosuppressive medications?
         1. Immediately off other immunosuppressive medications
         2. 1 month
         3. 2 months
         4. 3 months
         5. Other: _________________
   6. I would not alter the immunization schedule for INACTIVATED immunizations in patients with NS. They can receive them at any time.
   7. I would never provide an INACTIVATED immunization to a NS patient regardless of therapy or remission status.
   8. Other: _________________
2. What type of pneumococcal vaccine (PCV) do you recommend to NS patients? (**Circle all that apply**)
   1. 7-valent PCV
   2. 13-valent PCV
   3. 23-valent PCV
   4. I am not sure
   5. I do not recommend these vaccines
3. Is your office able to **administer** the pneumococcal vaccine?
   1. Yes (if yes, then which PCV vaccines are available to be administered in your office? **Circle all that apply**)
      1. 7-valent PCV
      2. 13-valent PCV
      3. 23-valent PCV
   2. No
   3. I am not sure
4. Do you communicate your recommendations for vaccination to the patient’s primary care provider?
   1. Yes (please specify mode of communication below and **Circle all that apply**)
      1. Letter
      2. Email
      3. Phone call
   2. No
   3. I am not sure
5. Would you recommend the 23-valent PCV to a NS patient if he/she had previously received the 13-valent or 7-valent PCV?
   1. Yes
   2. No
   3. I am not sure
6. Have you withheld **INACTIVATED** immunizations from NS patients when they are on any dose of steroids because they may not mount a response to the vaccine?
   1. Yes
   2. No
   3. I am not sure
7. Have you had a patient who acquired a vaccine-preventable disease in the absence of recommended vaccines?
   1. Yes
   2. No
   3. I am not sure
8. Do you have patients who still decline vaccines despite immunization education?
   1. Yes
      1. If yes, then what percent of NS patients refuse vaccines? ____________
      2. I am not sure
   2. No
   3. I am not sure
9. What else would you like to tell us about vaccine practices?

**Supplement 2**

Nephrotic Syndrome (NS) Immunization survey (PARENT/GUARDIAN SURVEY)

Today’s date: _____________________

DEMOGRAPHICS:

1. Where do you live (List the state)? ________________________________________________
2. Current age of **patient** (age in years): ______________________________________________
3. What is your **child’s** race?
   1. White
   2. Hispanic or Latino
   3. Black or African American
   4. Native American or American Indian
   5. Asian/Pacific Islander
   6. Other
4. Date of nephrotic syndrome diagnosis (List month and year): ___________________________
5. What is the name of the institution/practice where your child is receiving treatment for his/her nephrotic syndrome? _______________________
6. How many times has your child seen his/her pediatric nephrologist in the past year?
   1. Once
   2. 2-3 times
   3. 4 or more times
7. Which immunosuppressive medications has your child been given for his/her nephrotic syndrome? (**Circle all that apply**)
   1. Prednisone
   2. Tacrolimus (Prograf)
   3. Cyclosporine (Neoral, Gengraf, Sandimmune)
   4. Mycophenolate Mofetil (Cell Cept)
   5. Cyclophosphamide (Cytoxan)
   6. Rituximab
   7. Other: please specify _______________________
8. What is the highest degree or level of school that you **(parent/guardian)** have completed?
   1. No schooling completed
   2. Nursery school to 8^th^ grade
   3. Some high school
   4. High school graduate
   5. Some college
   6. Trade/technical/vocational training
   7. College graduate
   8. Some postgraduate work
   9. Post graduate degree
   10. I decline to answer
9. What is your household annual income?
   1. Under $25,000
   2. $25,000 - $39,999
   3. $40,000 - $49,999
   4. $50,000 - $74,999
   5. $75,000 - $99,999
   6. Over $100,000
   7. I decline to answer

IMMUNIZATIONS:

1. In the 4-6 weeks before your child had his/her first episode of nephrotic syndrome (NS), had your child received any of the immunizations on the front cover page of the survey?
   1. Yes
   2. No
   3. I am not sure.
2. Has your child received any immunizations since his/her initial diagnosis of NS?
   1. Yes
   2. No
   3. I am no sure.

If you answered “yes” to Question 11, then proceed to Question 12. Otherwise, skip to Question 13

1. Has your child ever experienced a NS relapse after receiving an immunization?
   1. Yes
      1. If yes, which immunization? **(Circle all that apply)**
         1. Varicella (chicken pox immunization) (VZV)
         2. Measles, mumps, rubella (MMR, Measles shot)
         3. Pneumococcal (PCV)
         4. Flumist (Nasal Influenza)
         5. Inactivated Influenza (Intramuscular injection or shot)
         6. Other: please specify ______________
   2. No.
   3. I am not sure.
2. Has your Nephrologist ever discussed which immunizations should be given to your child?
   1. Yes
   2. No
   3. I am not sure.

If you answered “Yes” to Question 13, then move onto Question 14. If you answered “No” or “I am not sure” to Question 13, then skip Question 14 and move onto Question 15.

1. Do you intend to immunize your child based on the recommended immunizations given by your Nephrologist?
   1. Yes
   2. No
      1. If no, what is the reason? _________________________________________
   3. I am not sure.

Please answer the following section pertaining to **LIVE** immunizations (ie MMR, Varicella (chicken pox shot), Flumist (Nasal influenza)): **Circle all that apply.**

1. When were you told that it was ok to receive **LIVE** immunizations?
   1. No one ever told me when it was ok to receive LIVE immunizations.
   2. When my child is off steroids and in remission.
   3. When my child is on low dose (every other day) steroids.
   4. When my child is on daily high dose steroids.
   5. When my child is off other immunosuppression medications (ie cyclosporine, tacrolimus, mycophenolate mofetil, cyclophosphamide, rituximab).
   6. I was told that my child could receive LIVE immunizations at any time regardless of type of medication or remission status.
   7. I was told that my child could never receive LIVE immunizations.
   8. Other: please specify ______________
   9. I am not sure.

Please answer the following section pertaining to **INACTIVATED** immunizations (ie pneumococcal (PCV) & inactivated influenza (intramuscular injection or shot)): **Circle all that apply.**

1. When were you told that it was ok to receive **INACTIVATED** immunizations?
   1. No one ever told me when it was ok to receive INACTIVATED immunizations.
   2. When my child is in remission and off steroids.
   3. When my child is on low dose (every other day) steroids.
   4. When my child is on daily high dose steroids.
   5. When my child is off other immunosuppression medications (ie cyclosporine, tacrolimus, mycophenolate mofetil, cyclophosphamide, rituximab).
   6. I was told that my child could receive INACTIVATED immunizations at any time regardless of type of medication or remission status.
   7. I was told that my child could never receive INACTIVATED vaccines.
   8. Other: please specify ______________
   9. I am not sure.
2. My **child** receives the **inhaled** flu (nasal, Flumist) vaccine:
   1. Every year in the last 5 years
   2. Occasionally
   3. None
3. My **child** receives the **flu shot** (intramuscular) vaccine:
   1. Every year in the last 5 years
   2. Occasionally
   3. None

16) My **family** (members living in the same household) receives the flu vaccine (Not counting the patient):

1. All family members
2. Some family members
3. None

17) Has your child ever received the pneumococcal immunization?

a. Yes (if yes, then please specify type of pneumococcal immunization below **(Circle all that**

**apply)**.

i. 7-valent pneumococcal vaccine

ii. 13-valent pneumococcal vaccine

iii. 23-valent pneumococcal vaccine

iv. I am not sure which pneumococcal vaccine was given

b. No

c. I am not sure.

18) Since your child’s NS diagnosis, has he/she been hospitalized for an infection that can be preventable by vaccines?

a. Yes (if yes, then please specify how many days your child was hospitalized below)

i. How many days was your child hospitalized? ______________

b. No

c. I am not sure.

19) Please include your vaccine record with the survey. (The study team will De-identify with study ID)

20) Please confirm that the vaccine record is complete or incomplete.

a. Complete

b. Incomplete

c. I am not sure.

21) What else do you want to tell us about how you feel about immunizations?

**Supplement 3**

Supplemental Table 1 - Immunization recommendations for children with nephrotic syndrome (NS) on immunosuppressive medications

| Vaccine category | Vaccines | Immunization recommendations in children with NS on commonly used immunosuppressive medications* | | | | |
| --- | --- | --- | --- | --- | --- | --- |
|  |  | Corticosteroids | Calcineurin inhibitor (CNI, i.e.- Tacrolimus, Cyclosporine) | Anti-proliferative agent (i.e.- Mycophenolate mofetil (MMF)) | Cytotoxic chemotherapy (i.e.- Cyclophosphamide (CTX)) | B-cell depleting agent (i.e.- Rituximab (RTX)) |
| Inactivated | Diphtheria | Inactive vaccines can be administered to patients on corticosteroid therapy.  Follow the ACIP schedule for inactivated vaccines without delay. | Inactive vaccines can be administered to NS patients on CNI. | Inactive vaccines can be administered to NS patients on MMF. | Inactive vaccines can be administered to NS patients on CTX. | Try to delay inactive vaccines (except influenza) for at least 6 months from the last dose of RTX.  If no finite endpoint of RTX, then administration of inactive vaccines may be considered with the recommendation to check vaccine titers when off of RTX therapy to ensure immunity. |
|  | *Haemophilus influenzae* type b |  |  |  |  |  |
|  | Hepatitis A |  |  |  |  |  |
|  | Hepatitis B |  |  |  |  |  |
|  | Human papilloma virus |  |  |  |  |  |
|  | Influenza (Inactive) |  |  |  |  |  |
|  | Meningococcal serogroups A, C, W, Y (MenACWY-CRM ≥2 months old, MenACWY-D ≥6 months old ) |  |  |  |  |  |
|  | Meningococcal serogroup B |  |  |  |  |  |
|  | Pertussis (acellular) |  |  |  |  |  |
|  | Pneumococcal conjugate (PCV 13) |  |  |  |  |  |
|  | Pneumococcal polysaccharide (PPSV 23) |  |  |  |  |  |
|  | Poliovirus (inactivated) |  |  |  |  |  |
|  | Tetanus |  |  |  |  |  |
| Live attenuated | Influenza (Live attenuated) | Live vaccines should not be given when on high-dose steroids (dose of either ≥2 mg/kg or ≥20 mg/day of prednisone for ≥14 days).  May be given when 1) off high dose steroids for at least 1 month; 2) on short term steroids (<14 days); 3) on low dose steroids (<20 mg/day or <2mg/kg/day); 4) on long-term, alternate-day treatment with short-acting steroids; or 5) on topical, inhaled, or intra-articular steroid injection. | Live vaccines should not be given when on CNI.  May be given when off of CNI for at least 2 months. | Live vaccines should not be given when on MMF.  May be given when off of CNI for at least 2 months. | Live vaccines should not be given when on CTX.  May be given when off of CTX for at least 3 months. | Live vaccines should not be given when on RTX.  May be given when off of RTX for at least 6 months. |
|  | Measles, Mumps, Rubella |  |  |  |  |  |
|  | Rotavirus |  |  |  |  |  |
|  | Varicella |  |  |  |  |  |

* Administer when age-eligible as per ACIP guidelines for children and adolescents with altered immune competence.

Abbreviations: ACIP, Advisory Committee on Immunization Practices

**Supplement 4**

Supplemental Table 2 - Provider PPSV 23 responses by region. Data are shown as number (percent).

|  | Total  N=50 (%) | Coastal  N=12 (%) | Midwest  N=21 (%) | South  N=17 (%) | Fischer’s exact *p* |
| --- | --- | --- | --- | --- | --- |
| How often do you/your staff review immunization records at initial evaluation of patients with NS?) | | | | | 0.31 |
| 1: Never | 1 (2) | 0 (0) | 0 (0) | 1 (6) |  |
| 2: Rarely | 9 (18) | 4 (33) | 2 (10) | 3 (18) |  |
| 3: Sometimes | 9 (18) | 3 (25) | 3 (14) | 3 (18) |  |
| 4: Often | 15 (30) | 1 (8) | 10 (48) | 4 (24) |  |
| 5: Always | 14 (28) | 3 (25) | 5 (24) | 6 (35) |  |
| 6: I am not sure | 2 (4) | 1 (8) | 1 (5) | 0 (0) |  |
| How often do you/your staff review immunization records on a periodic basis (annually) with your NS patients?) | | | | | <0.01 |
| 1: Never | 2 (4) | 0 (0) | 1 (5) | 1 (6) |  |
| 2: Rarely | 8 (16) | 7 (58) | 0 (0) | 1 (6) |  |
| 3: Sometimes | 11 (22) | 3 (25) | 6 (29) | 2 (12) |  |
| 4: Often | 17 (34) | 0 (0) | 9 (43) | 8 (47) |  |
| 5: Always | 12 (24) | 2 (17) | 5 (24) | 5 (29) |  |
| 6: I am not sure | 0 (0) | 0 (0) | 0 (0) | 0 (0) |  |
| Would you recommend the PPSV 23 to a NS patient if he/she had previously received the 13-valent or 7-valent PCV?) | | | | | 0.03 |
| 1: Yes | 46 (92) | 9 (75) | 21 (100) | 16 (94) |  |
| 2: No | 0 (0) | 0 (0) | 0 (0) | 0 (0) |  |
| 3: I am not sure | 4 (8) | 3 (25) | 0 (0) | 1 (6) |  |

Abbreviations: NS, nephrotic syndrome; PPSV 23, pneumococcal polysaccharide vaccine 23-valent; PCV, Pneumococcal conjugate vaccine
